# Age-related differences in polyfunctional T cell responses

**DOI:** 10.1186/1742-4933-11-14

**Published:** 2014-10-23

**Authors:** Puja Van Epps, Richard Banks, Htin Aung, Michael R Betts, David H Canaday

**Affiliations:** 1 Geriatric Research Center Clinical Core (GRECC), Louis Stokes Cleveland VA Medical Center, 10701 East Blvd, Cleveland, Ohio 44106, USA; 2 Department of Microbiology, Perelman School of Medicine, University of Pennsylvania, Philadelphia, Pennsylvania 19104, USA; 3 Division of Infectious Diseases, Case Western Reserve University School of Medicine, 10900 Euclid Ave, BRB 1022, Cleveland, Ohio, 44106-4684, USA

**Keywords:** Aging, T cells, Polyfunctionality, Immunesenescence

## Abstract

**Background:**

A reduced number of naïve T cells along with an accumulation of differentiated cell types in aging have been described but little is known about the polyfunctionality of the T cell responses. In this study we compared the individual and polyfunctional expression of IFN-γ, MIP-1α, TNF-α, perforin, and IL-2 by T cell subsets, including the newly described stem cell like memory T cells (T_SCM_), in response to stimulation with superantigen staphylococcal enterotoxin B (SEB) in older (median age 80, n = 23) versus younger (median age 27; n = 23) adults.

**Results:**

Older age was associated with a markedly lower frequency of CD8+ naïve T cells (11% vs. 47%; p < 0.0001) and an expansion in memory T cell subsets including central memory (p < 0.05), effector memory and effector T cells (p < 0.001 for both). There was also a decline in CD4+ naïve T cells in older subjects (33% vs. 45%; p = 0.02). There were no differences in frequencies or polyfunctional profiles of T_SCM_ between groups. CD8+ naïve cells in the older group had increased expression of all functional parameters measured compared to the younger subjects and exhibited greater polyfunctionality (p = 0.04). CD4+ naïve T cells in the older group also showed greater polyfunctionality with a TNF-α and IL-2 predominance (p = 0.005). CD8+ effector memory and effector T cells exhibited increased polyfunctionality in the older group compared with younger (p = 0.01 and p = 0.003).

**Conclusions:**

These data suggest that aging does not have a negative effect on polyfunctionality and therefore this is likely not a major contributor to the immunesenescence described with aging.

## Background

Decline in immune function with age or *immunesenescence* is a well-described phenomena [1,2]. Impaired cell-mediated immunity in older adults renders them increasingly susceptible to infectious diseases while at the same time leading to diminished immune responses following vaccination, the very strategy used to prevent infections [3,4]. Increased morbidity and mortality related to influenza along with a weakened immune response to influenza vaccine in the elderly have served as a prime example of this phenomena [5,6]. In part immunesenescence is related to the changing phenotypic composition of T cells with age. Studies demonstrate a decline in naïve T cells along with an accumulation of late differentiated T cell types in aging, particularly in CD8+ T cells [7,8].

The focus of studies on cell-mediated immunity in aging has been either on antigen specific vaccine or viral responses. While a robust humoral response to influenza is necessary to prevent primary infection, eventual viral clearance is dependent on the presence of influenza-specific CD8+ T cells [9]. Studies have demonstrated diminished expression of IFN-γ and granzyme B in CD8+ T-cell subsets in older adults vaccinated with influenza [10]. Similarly, diminished age related T cell responses to immunization with hepatitis B surface antigen have previously been demonstrated [11]. Similar studies with regards to viral specific T cell immunity such as RSV-specific responses have been reported to be deficient in older age [12,13]. There exists however a paucity of data with regard to polyfunctional capabilities of T cells in aging and how it relates to the changing phenotype.

Multiparameter flow analysis has offered the ability to simultaneously analyze multiple functional responses at the individual cell level. Use of such analysis particularly in the field of HIV-1 has advanced our knowledge of polyfunctionality of T cells. While enhanced polyfunctionality in HIV-1- specific CD8+ T cells has been associated with superior control of the virus in some studies, others have not found this to be an important factor in viral control [14-16]. Questions remain whether highly functional T cells actually confer increased immune protection. We have previously demonstrated that age does not impact the polyfunctionality of memory CD8+ T cells in response to acute and chronic viral infections [17]. To our knowledge such analysis has not been utilized to study the effect of age on polyfunctional signatures of the full repertoire of CD8+ and CD4+ T cell subtypes. In this study we sought to determine the effect of age on individual functional parameters and polyfunctional capabilities of both CD4+ and CD8+ T cell subsets, including naïve and recently described T_SCM_ in response to stimulation with the superantigen SEB.

## Results

Twenty three subjects were analyzed in each age group. Baseline characteristics of the study participants are listed in Table 1. The older group had a median age of 80 years ranging from 68–91 while the median age of the younger group was 27, ranging from 23–36. There were no significant gender or race differences between groups.

**Table 1:** Baseline characteristics of subjects

	**Older n = 23**	**Younger n = 23**	**P value**
Median age (range)	80 (68–91)	27 (23–36)	<0.001
Gender (%)	Females 5 (22)	Females 8 (35)	0.5
	Males 18 (78)	Males 15 (65)	
Race	Black 7 (30%)	Black 8 (34%)	0.9
	White/other 16 (70%)	White/other 15 (65%)	

### Age associated decline in naïve T cells with increased expression of functional parameters

**Figure 1:**
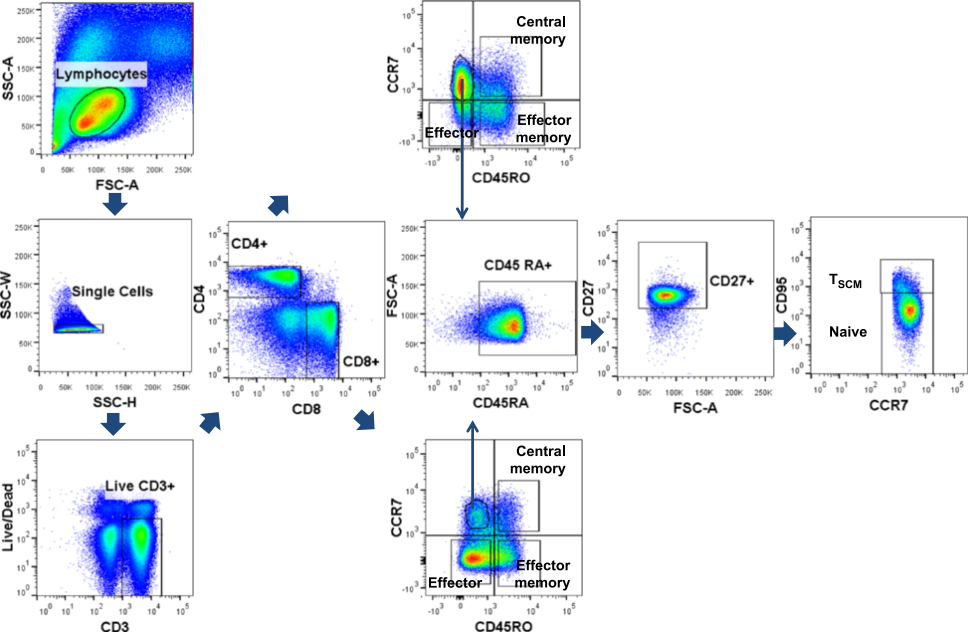
**Flow cytometry gating scheme.** Dot plots of a representative subject are shown.

**Figure 2:**
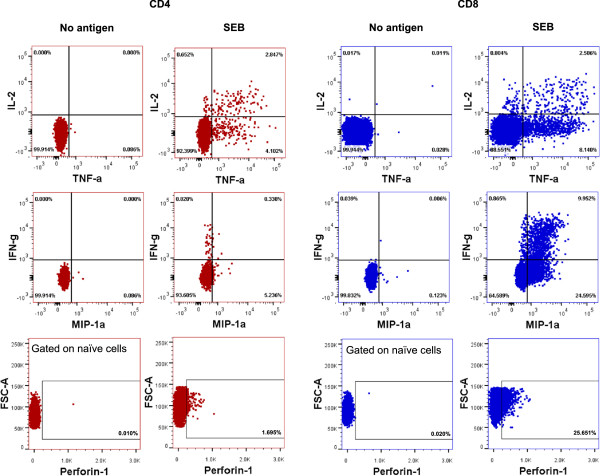
**Multiparameter intracellular cytokine gating scheme.** Dot plots of a representative subject are depicted. Effector memory subtype is shown. Unstimulated samples were used to create the gates for all parameters except perforin. Since there is constitutive expression of perforin, unstimulated naïve cells due to their extremely low perforin expression were used to define the perforin gate.

**Figure 3:**
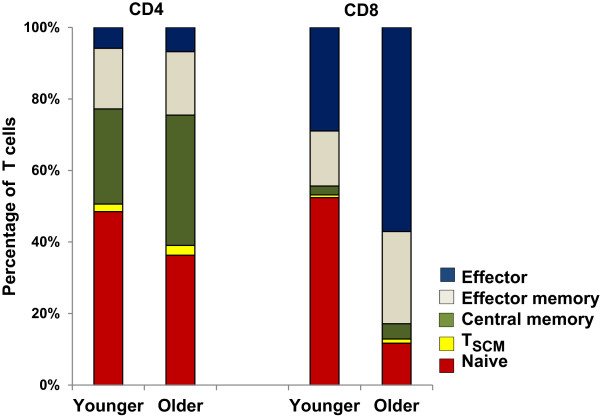
**Distribution of T cell subsets among CD4+ and CD8+ T cells.** Median values of T cell subsets frequencies among the young and old groups are represented by horizontal bars.

First we compared T cell subset frequencies between younger and older groups. Flow cytometry gating strategies employed throughout this study are depicted in Figures 1 and 2. As expected, older age was associated with decreased percentage of naïve subtype among both CD4+ and CD8+ T cells though the decline was much more pronounced in the latter (CD4+: 45% vs. 33%, p = 0.02; CD8+: 47% vs. 11%, p < 0.0001 for younger and older, respectively; Figure 3). The CD4+ central memory (CM) subset was increased in older compared with younger subjects (33% vs. 24%, p = 0.01). The proportions of CD8+ CM, effector memory (EM) and effector subtypes were also greater in older subjects compared with younger (p = 0.01 for CM, p < 0.001 for the rest; Figure 3).

**Figure 4:**
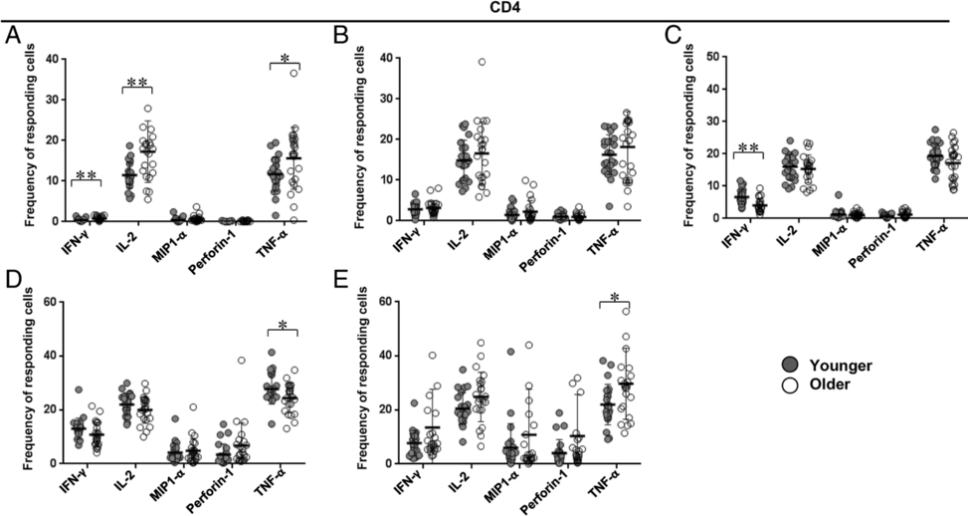
**Frequencies of CD4+ T cell subsets responsive to individual functional parameters in younger vs. older subjects. A**. naïve. **B**. T_SCM._**C**. central memory. **D**. effector memory **E**. effector. Significant P values marked as *p < 0.05; **p < 0.01; ***p < 0.001. Bars represent medians, error bars represent interquartile ranges. Filled circles represent younger and open circles represent older subjects.

**Figure 5:**
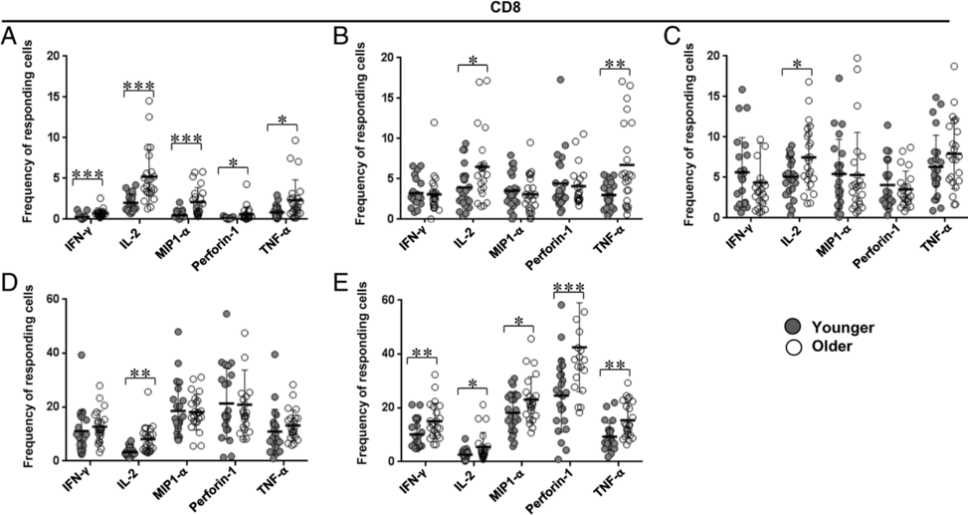
**Frequencies of CD8+ T cell subsets responsive to individual functional parameter in younger vs. older subjects. A**. naïve. **B**. T_SCM_. **C**. central memory. **D**. effector memory **E**. effector. Significant P values marked as *p < 0.05; **p < 0.01; ***p < 0.001. Bars represent medians, error bars represent interquartile ranges. Filled circles represent younger and open circles represent older subjects.

In terms of functional activity of the T cell subsets, while there were several significant differences in frequencies of cells expressing each parameter between the two groups, the differences were much more pronounced among the CD8+ T cells (Figures 4 and 5). There was increased expression of all five functional parameters within the CD8+ effector subset in the older group (Figure 5E). Similarly within the CD8+ naïve subset, older subjects had higher frequency of all parameters, though the overall frequency except for IL-2 and TNF-α was quite small (Figure 5A). In the older group, IL-2 expressing cells were increased within CD8+ T_SCM_, CM, EM cells (Figures 5B-D). Among the CD4+ naïve T cells, again there was an increase in frequency of IFN-γ, IL-2, and TNF-α cytokine expressing cells in the older subjects, though the overall expression of IFN-γ was low in both groups (Figure 4A). Frequency of TNF-α expressing cells was also increased among the CD4+ EM and effector subtypes in the older subjects (Figures 4D-E). Of all the functional parameters, IFN-γ expressing cells within the CD4+ CM subset were the only ones decreased in the older subjects compared to younger ones (Figure 4C).

### Polyfunctional profile of T cell subsets

**Figure 6:**
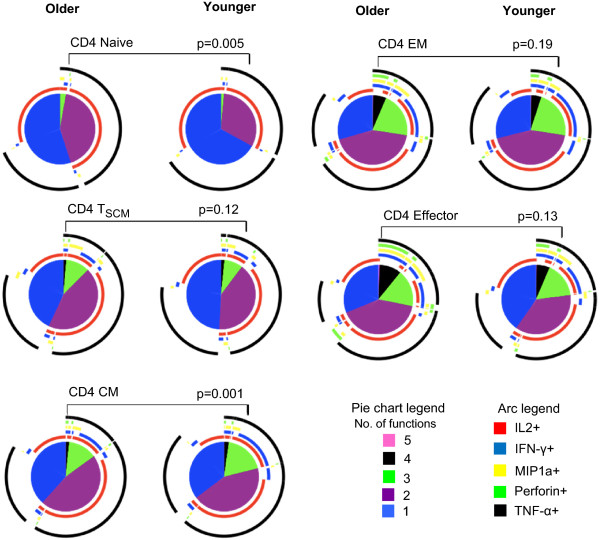
**Comparison of polyfunctional profiles of older vs. younger subjects among the various CD4+ T cell subsets.** Pies compare the proportion of SEB-responding CD4+ T cells in younger vs. older subjects with distinct functional pattern for various subsets. The arc around each pie represents the individual functional parameter as noted in the Arc legend. The pie slice color which represent number of functions the cell performs ranging from 1 to 5 are noted in the pie chart legend. Statistical comparisons between the pies were performed using Monte Carlo permutation analysis by SPICE.

**Figure 7:**
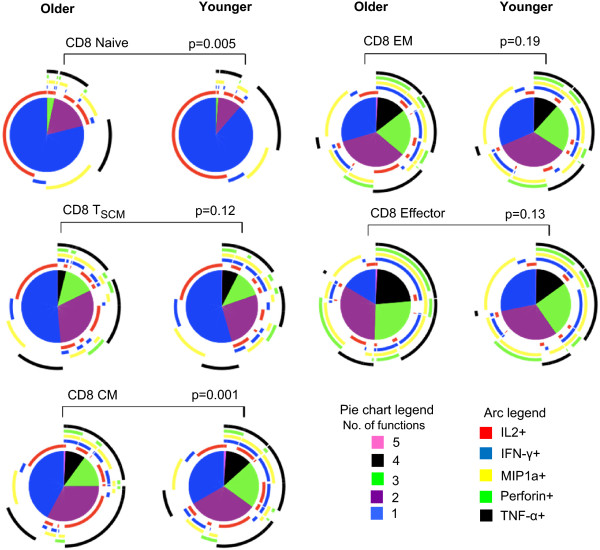
**Comparison of polyfunctional profiles of older vs. younger subjects among the various CD8+ T cell subsets.** Pies compare the proportion of SEB-responding CD8+ T cells in younger vs. older subjects with distinct functional pattern for various subsets. The arc around each pie represents the individual functional parameter as noted in the Arc legend. The pie slice color which represent number of functions the cell performs ranging from 1 to 5 are noted in the pie chart legend. Statistical comparisons between the pies were performed using Monte Carlo permutation analysis by SPICE.

**Figure 8:**
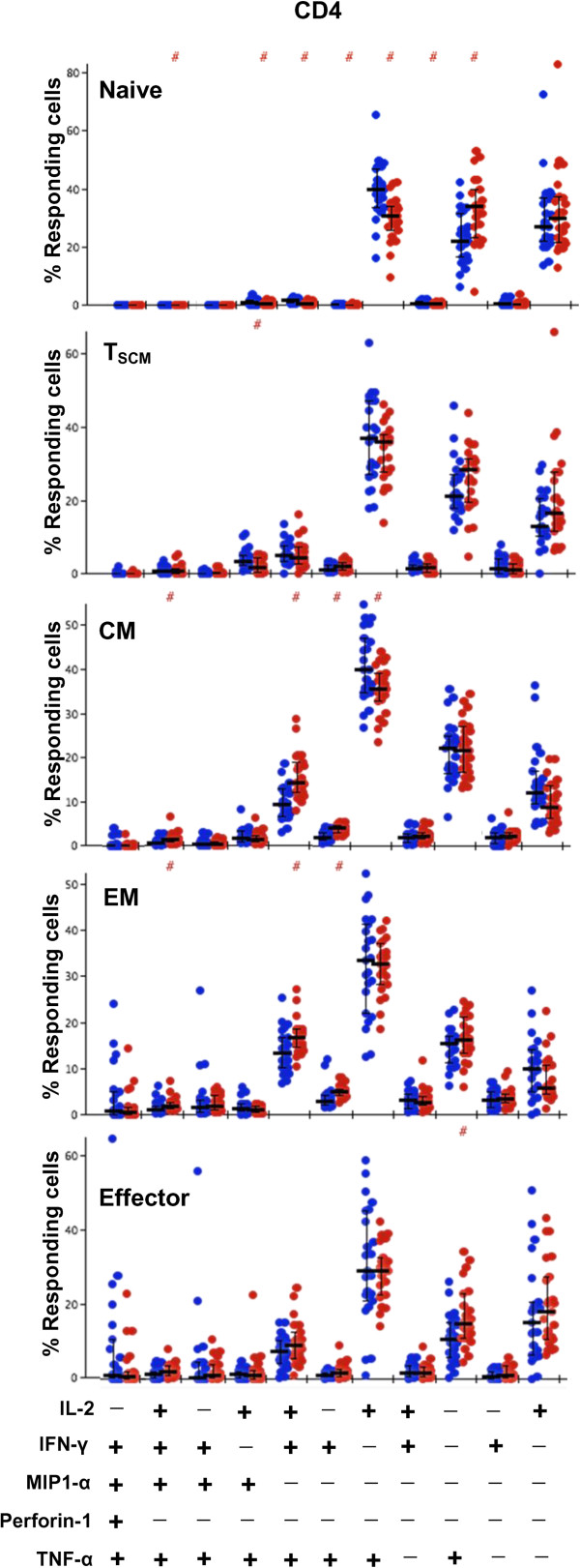
**Frequencies of CD4+ T cell subsets responsive to functional parameter combinations in younger vs. older subjects.** The bar graphs depict the proportion of SEB-responding CD4+ T cells in younger (red circles) and older (blue circles) subjects with distinct functional pattern for all subsets. Bars represent medians, error bars represent interquartile ranges. The bar graph shows the individual subjects’ proportions for polyfunctional combinations. Only combinations with 5% or greater response frequency in at least one subset are depicted for clarity. A # at the top of the bar graph indicates a statistically significant difference (p < 0.05) of that individual functional combination between younger and older subjects as determined by Wilcoxon Rank Sum test.

**Figure 9:**
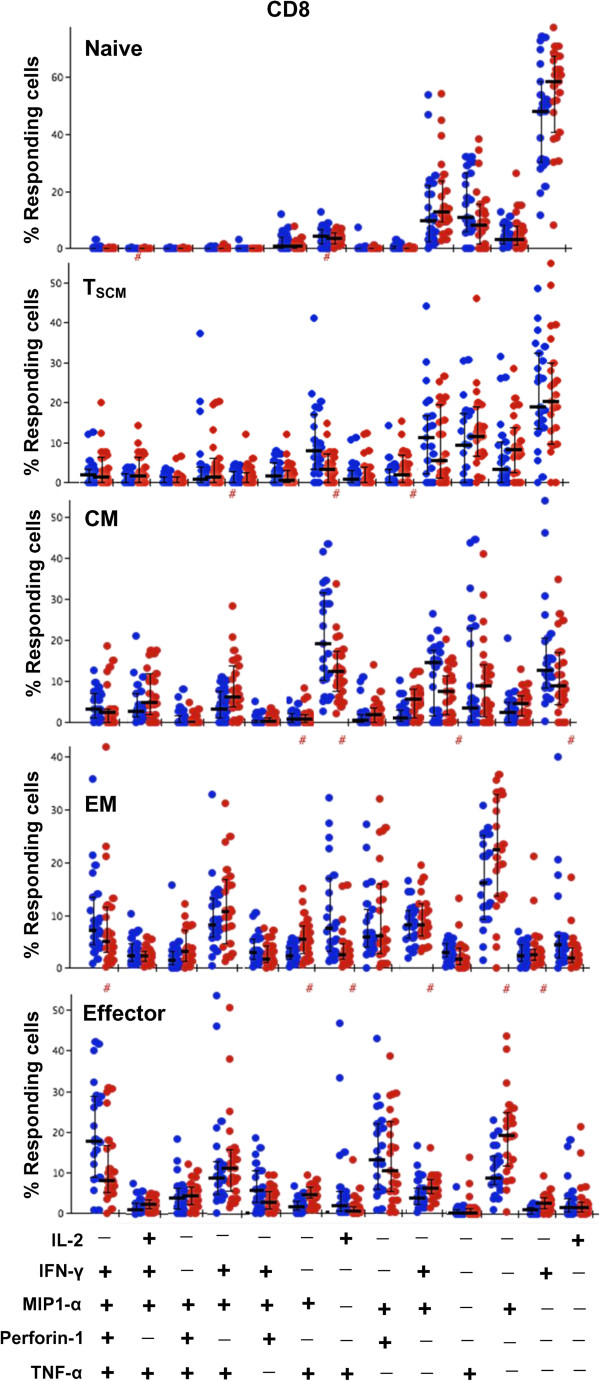
**Frequencies of CD8+ T cell subtypes responsive to functional parameter combinations in younger vs. older subjects.** The bar graphs depict the proportion of SEB-responding CD8+ T cells in younger (red circles) and older (blue circles) subjects with distinct functional pattern for all subsets. Bars represent medians, error bars represent interquartile ranges. The bar graph shows the individual subjects’ proportions for polyfunctional combinations. Only combinations with 5% or greater response frequency in at least one subset are depicted for clarity. A # at the top of the bar graph indicates a statistically significant difference (p < 0.05) of that individual functional combination between younger and older subjects as determined by Wilcoxon Rank Sum test.

Next we compared polyfunctional profiles of all the CD4+ and CD8+ T cell subtypes between the younger and older subjects. Among the CD4+ and CD8+ naïve T cells, while highly polyfunctional cells were infrequent in both groups, the polyfunctional profiles differed significantly between groups (p = 0.005 and p = 0.04 for CD4+ and CD8+ respectively; Figures 6 and 7). Older subjects had more triple and dual functional cells compared with younger subjects. Triple functional IL-2 + MIP-1α + TNF-α + cells were increased in both CD4+ and CD8+ naïve T cells in the older group compared with younger subjects (Figures 8 and 9). While both IL-2 and TNF-α were dominant among the CD4+ naïve T cells, IL-2 only expressing cells were the dominant phenotype in both groups among the CD8+ naïve T cells. Though the overall differences were small, CD8+ EM and effector cell types both exhibited increase in polyfunctionality in the older subjects compared with younger (p = 0.01and p = 0.003 for EM and effector respectively; Figure 7). Among the older group, highly polyfunctional CD8+ effector T cells expressing 5 or 4 parameters were significantly increased compared with the younger group (Figure 9). Both CD4+ and CD8+ CM subtype exhibited differences in polyfunctional profile between the two groups (p = 0.001 and p = 0.01; Figures 6 and 7). Among the CD4+ CM cells, IL-2 + INFγ + TNF-α + phenotype was more frequent among the younger subjects, while the IL-2 + TNF-α + phenotype was more frequent in the older group (Figure 8). Similarly among the CD8+ CM cells while IL-2 + TNF-α + phenotype was more frequent in older subjects, triple function cells were more common in the younger subjects (Figure 9). There were no other significant age-related differences in polyfunctional profiles of other CD4+ subtypes.

As a superantigen SEB directly stimulates T cells by cross-linking major histocompatibility complex class II molecules on APC with the variable portion of the T-cell antigen receptor of specific β chains [18]. To ensure that any observed differences between groups were not attributable to differing Vβ distribution of T cell receptors (TCR), Vβ distribution analysis was performed on a subset of subjects from each age group and was not found to be different between groups across all the T cell subsets (data not shown).

## Discussion

In the present study we sought to determine the effect of age on polyfunctional profiles of T cell subtypes. We observed a decline in both CD4+ and CD8+ naïve T cell frequencies in older adults which was accompanied by an unexpected enhanced functionality in this subset. Though proportionally lower in older adults, the naïve T cells displayed augmented functional capabilities, both at the individual functional parameter as well as polyfunctional level. Similarly, polyfunctionality was slightly enhanced within the EM and effector CD8+ T cell subsets in the older group. CM subsets of both CD4+ and CD8+ T cells were increased in the older group however there was an age-associated decline in polyfunctionality in this subset. Polyfunctional profiles of the remaining CD4+ and CD8+ T cell subsets were not influenced by age.

While the observed shift in T cell subset phenotype from naïve to differentiated memory cell types with age is not unexpected, the effect of age on polyfunctional profile of T cells subsets has not previously been described. Decreased ability to respond effectively to neoantigens is one of the hallmarks of immunesenescence. However, based on our findings, naïve T cells in older individuals are capable of responding provided sufficient or the appropriate stimulus is present. The key may be the reduced numbers and repertoire of the naive T cells rather than their lack of functional ability.

CD8+ effector T cells were markedly increased in older subjects and also exhibited increased expression of all functional parameters along with increased polyfunctionality in response to SEB stimulation. Recently, CMV seropositivity has been associated with increased levels of polyfunctional CD8+ T cells in response to stimulation with SEB in young and middle age subjects [19]. The increase in polyfunctionality was found to be due to a CD8 + CD57+ T cell expansion in CMV-seropositive individuals and was independent of age. Since we did not specifically examine CMV seropositivity of subjects we cannot make any conclusions regarding this as possible mechanism for increased polyfunctionality of memory CD8+ T cells. Similarly, age-related changes in the human bone marrow were not found to impair the maintenance of a high number of polyfunctional memory CD4+ and CD8+ T cells in the bone marrow of elderly individuals [20]. We have previously demonstrated that West Nile virus, CMV and Epstein Bar virus specific CD8+ T cell polyfunctionality did not differ with age [17]. While increased or preserved polyfunctionality of effector CD8+ T cells may mean immunesenescence is likely not a result of a lack of ability to respond in the presence of the appropriate antigenic stimulus, it may represent a heightened state of pro-inflammatory cytokine production in older adults. Chronic CMV infection is thought to be a major driving force of immunosenescence and has been shown to impair T cell functionality in response to antigens such as the influenza vaccine [21,22]. There may also be a role of lack of specific antigenic boost with aging resulting in poor maintenance of protective immunity such as the decline in VZV-specific memory with age [23,24]. One limitation of the study is that the responses studied are not antigen specific and therefore do not give insight into cell-mediated immunity with regard to any particular disease state or antigen. However, responses to non-specific stimuli such as SEB do provide a general snapshot of T cell functionality in aging. Also SEB signals through the TCR rather than other mitogens such as PMA and Ionomycin that bypass this step.

## Conclusions

In summary, the present study demonstrates enhanced polyfunctionality of naïve T cells in older adults. This is particularly true in CD8+ T cells and contrasted with sharp decline in proportion of this subset with age. We also demonstrated enhanced functionality of EM and effector CD8+ T cells in this group. CD8+ T cells are a critical arm of cell-mediated immunity against pathogens and the age-related differences we have observed help further our understanding of immunesenescence. Whether this enhanced functional expression in aging represents preserved functionality in the presence of specific antigenic stimulus or a state of pro-inflammatory milieu requires further investigation.

## Methods

### Study subjects

Experiments were conducted using blood from healthy volunteers after obtaining informed consent and approval by the Case Western Reserve University and Louis Stokes Cleveland VA Medical Center institutional review board. Samples were divided into two groups based on age, younger (21–36 years) and older (≥65 years).`

### Cell stimulation and staining

Peripheral blood mononuclear cells (PBMC) were isolated from blood samples by Ficoll Plaque Plus (GE Healthcare/Amersham Biosciences) density gradient centrifugation according to the manufacturer’s instructions. PBMC were then cryopreserved in 90% fetal bovine serum and 10% DMSO till analysis. Cryopreserved PBMC were thawed and re-suspended in X-Vivo-15 serum-free media (Lonza). Between 0.5-1 × 10^6^ PBMC/tube were stimulated with sub-maximal concentration of SEB (SIGMA) at 0.1 μg/ml in the presence of anti-CD28/49d (1 μg/ml each, eBioscience and Biolegend), and brefeldin A (SIGMA). Each sample had a corresponding negative control tube without SEB. Following overnight stimulation a 13-color flow cytometry assay that simultaneously determined responses of five functions and identified memory and naive CD8+ and CD4+ T cell subsets was performed. PBMC were washed with PBS and stained with anti-CCR7-PE/Cy7 (BD) for 15 min at 37°C and followed by live-dead yellow, anti-CD45RA-PE-TR, CD27-Qdot605 (all Invitrogen), CD45RO-BV711, CD95-PE/Cy5, CD4-APC/Cy7 and CD8-BV510 (all Biolegend) at room temperature for 20 minutes. Cytofix/Perm (BD) wash reagents were used for intracellular staining according to the manufacturer’s instructions with CD3-BU395 (BD Bioscience), anti-IFN-γ-Alexa700, IL-2APC, TNF-α-PacBlue (all Biolegend), MIP-1α-FITC (R & D Systems), and perforin-1-PE (clone B-D48, Cell Science). Cells were then washed and fixed in 2% paraformaldehyde.

### Flow cytometry

Data were acquired on LSRII flow cytometer (BD Biosciences) and analyzed using FlowJo 7.6.4 software (Tree Star, Ashland, OR). Lymphocytes were identified using forward and side scatter, and further gated to include only singlet events and live cells (Figure 1). CD3+ cells were subsequently gated to determine CD4+ and CD8+ subsets: naïve (CD45RO- CCR7+ CD45RA + CD27+ CD95-), T_SCM_ (CD45RO- CCR7+ CD45RA + CD27+ CD95+), CM (CD45RO + CCR7+), EM (CD45RO + CCR7-), effector (CD45RO- CCR7-). We used CD95 as a marker for T_SCM_ within the naïve subset adapted from the gating strategy described by Klatt et al. and Lugli et al. [25,26]. Intracellular gating of the functional parameters with and without SEB is demonstrated in Figure 2 from a representative subject. SEB specific Vβ distribution analyses were performed on a subset of subjects (n = 16; 8 from each age group) with FITC (Vβ 3) (eBioscience) or PE (Vβ 12, 13.2, 14, 17, 20) (all BD Biosciences) conjugated anti-Vβ antibodies which are known to interact with SEB [18,27].

### Statistical analysis

All data except for perforin responses were background-subtracted using the un-stimulated samples. Absolute perforin data were used for analysis to take into account constitutive perforin expression. The gating for perforin was based on the naïve cells having very low perforin expression. Statistical analysis was performed using GraphPad Prism 6. Statistical significance was defined as *p* < 0.05 as calculated using Mann–Whitney Rank Sum test. Boolean analysis in Flow Jo was used to assess polyfunctionality. SPICE 5.3 (National Institutes of Health) software was used to graphically depict polyfunction and compare using Monte Carlo permutation analysis the profiles between groups.

## Abbreviations

T_SCM_: Stem cell like memory T cells; CM: Central memory; EM: Effector memory; TCR: T cell receptors; PBMC: Peripheral blood mononuclear cells; SEB: Staphlococcal enterotoxin B.

## Competing interests

The authors declare that they have no competing interest.

## Authors’ contributions

PVE, DHC, and MRB participated in the design and analysis of the study. PVE and HA carried out immunological assays, flow analysis, and flow presentation. RB was the clinical coordinator for screening, recruitment, and processing of the samples. PVE and DHC wrote the manuscript and all authors approved the final manuscript.
